# Functional Soy and Lupin Protein-Based Beverages Modulate Gut Microbiome and Attenuate Metabolic Dysregulation in Adolescent Boys with Overweight and Obesity

**DOI:** 10.3390/nu18132049

**Published:** 2026-06-23

**Authors:** Tereso J. Guzmán, Lucila A. Godínez-Méndez, Irma C. Soto-Luna, Vidal Delgado-Rizo, Pedro M. García-López, Enrique Romero-Velarde, Belinda Vargas-Guerrero, Israel Hurtado-Díaz, Adriana M. Salazar-Montes, Carmen M. Gurrola-Díaz

**Affiliations:** 1 Instituto de Investigación en Enfermedades Crónico-Degenerativas/Instituto Transdisciplinar de Investigación e Innovación en Salud, Departamento de Biología Molecular y Genómica, Centro Universitario de Ciencias de la Salud, Universidad de Guadalajara, Guadalajara C.P. 44340, Jalisco, Mexico; goozman57@hotmail.com (T.J.G.); catalina.soto@academicos.udg.mx (I.C.S.-L.); belinda.vargas@academicos.udg.mx (B.V.-G.); israel.hurtado@academicos.udg.mx (I.H.-D.); adriana.smontes@academicos.udg.mx (A.M.S.-M.); 2 Departamento Académico de Ciencias Básicas, Universidad Autónoma de Guadalajara. Av. Patria 1201, Zapopan C.P. 45129, Jalisco, Mexico; lucila.godinez@edu.uag.mx; 3 Centro de Investigación en Inmunología y Dermatología (CIINDE), Departamento de Fisiología, Centro Universitario de Ciencias de la Salud, Universidad de Guadalajara, Guadalajara C.P. 45190, Jalisco, Mexico; vidal.delgado@academicos.udg.mx; 4 Departamento de Botánica y Zoología, Centro Universitario de Ciencias Biológicas y Agropecuarias, Universidad de Guadalajara, Zapopan C.P. 45200, Jalisco, Mexico; macedonio.garcia@academicos.udg.mx; 5 Instituto de Nutrición Humana, División de Pediatría, Hospital Civil de Guadalajara “Dr. Juan I. Menchaca”, Universidad de Guadalajara, Guadalajara C.P. 44340, Jalisco, Mexico; enrique.rvelarde@academicos.udg.mx

**Keywords:** plant proteins, incretins, insulin sensitivity, gut microbiota

## Abstract

**Background/Objectives**: Given the rising prevalence of overweight and obesity in pediatric populations, identifying effective nutritional interventions for metabolic management is crucial. Beyond their nutritional value, soy and lupin proteins are recognized for their bioactive properties. We formulated two protein-enriched functional beverages and evaluated their impact on the metabolic profile and gut microbiota of adolescent boys with overweight or obesity. **Methods**: A randomized, double-blind clinical trial was conducted with 30 Mexican male adolescents (12–16 years old). Participants were randomly assigned to consume a functional beverage providing a daily 10 g portion of either soy or lupin protein for 5 weeks. **Results**: Following the intervention, both groups exhibited significantly attenuated fasting glucose (soy: 93.1 vs. 99.5 mg/dL; lupin: 92.3 vs. 97.9 mg/dL) and C-peptide levels. Consequently, insulin sensitivity, assessed via the HOMA2 index, improved significantly in both cohorts. The soy protein group showed a marked reduction in total cholesterol (–10.4%) and triglycerides (–17.1%). Furthermore, serum levels of plasminogen activator inhibitor-1 (PAI-1) and visfatin were decreased after both interventions. A post-treatment reduction in glucose-dependent insulinotropic polypeptide (GIP) was specifically observed in the lupin group. Regarding the gut microbiota, both protein-based beverage interventions correlated with enhanced 16S rDNA diversity and increased the abundance of the *Bacillota* phylum and butyryl-CoA transferase-positive bacteria. **Conclusions**: Our data suggests that the daily consumption of soy or lupin protein-based beverages could exert beneficial metabolic and endocrine effects in adolescent boys with overweight and obesity, potentially mediated by the modulation of the gut microbiome.

## 1. Introduction

Obesity is now recognized as a chronic disease and considered an important risk factor for further development of cardiovascular and metabolic conditions. Particularly relevant, individuals with obesity during puberty and adolescence are more likely to carry it into adulthood [1]. Adolescents with obesity often present at least one biochemical or clinical cardiovascular risk factor, with over one-quarter exhibiting more than two factors [2]. These facts highlight the current need for establishing early-stage interventions that counteract the burden of these conditions [2,3].

The management of obesity in children and adolescents focuses on encouraging lifestyle changes, including modifications on dietary patterns, as well as fostering health behaviors and promoting healthy living [4,5]. There is a growing interest in incorporating functional foods into the diet that, beyond providing basic nutrition, exert significant biological effects capable of modulating anthropometric, biochemical, and metabolic parameters [6]. In this regard, accumulating evidence suggests that the integration of specific bioactive dietary components can mitigate the metabolic complications associated with obesity.

Legumes represent a sustainable alternative to animal-derived proteins; in particular, soy and lupin stand out due to their high protein content and the health-promoting effects attributed to their bioactive protein fractions [7,8]. Consequently, both have been extensively incorporated into a wide variety of food products as functional ingredients [6,9,10]. Soy consumption has been associated with the prevention of chronic diseases such as cancer, obesity, and atherosclerosis. Likewise, it purportedly exerts protective effects on the cardiovascular system, lowering blood pressure and having antithrombotic properties. Besides these effects, fermented soy products like tempeh have been shown to induce a higher response to hormones that regulate appetite in obese women [11]. Similar to adults, clinical research in children or adolescents shows that soy protein reduces lipid levels [12]. Finally, epidemiological evidence suggests that soy consumption is linked to significant health benefits, including a decreased incidence of obesity and type 2 diabetes, primarily through the modulation of lipid and glucose metabolism [13].

On the other hand, recent evidence highlights *Lupinus angustifolius* as a potent source of bioactive compounds, particularly protein hydrolysates and peptides, which exert anti-inflammatory, antioxidant, and anti-diabetic activities, positioning lupin as a high-value functional ingredient for metabolic health [14]. Clinical studies have evaluated the impact of lupin proteins on body weight and satiety, and indicate beneficial effects on blood pressure, glycemia, and lipid serum levels. Moreover, it has been demonstrated that lupin may reduce appetite and consequently influence the maintenance of a normal body weight in humans. In a study conducted on healthy adults, it was observed that a breakfast with lupin flour provided higher levels of satiety and lower energy intake compared to a control meal [15,16,17].

Regarding their influence on glucose homeostasis, the hypoglycemic effects in both legumes have been assessed separately. Soy as well as lupin-based products reduce glycemia in healthy adults [16,18] and patients with type 2 diabetes [19]. Interestingly, the evidence suggests that a healthy diet and lifestyle in the early stages of life reduce the probability of developing chronic diseases such as type 2 diabetes mellitus or cardiovascular disease. Additionally, nutritional supplementation with legume protein-based products, such as soy or lupin, may exert immediate health benefits and yield long-term results when consumed before or during adolescence.

On the other hand, recent studies have established a significant link between obesity and gut microbiota, demonstrating that shifts in microbial diversity and richness are closely associated with metabolic outcomes [20]. The ‘Western diet’, characterized by a high intake of saturated fats and refined sugars, plays a pivotal role in inducing intestinal dysbiosis [21,22]. In both murine models and individuals with obesity, the primary taxonomic shifts involve an altered *Bacillota*/*Bacteroidota* ratio, typically manifested as an expansion of *Bacillota* and a concomitant reduction in *Bacteroidota*. Within the *Bacillota* phylum, these changes are frequently characterized by an increase in the genera *Staphylococcus* and *Lactobacillus*, alongside a depletion of butyrate-producing bacteria [23,24,25]. Furthermore, a notable reduction in *Akkermansia muciniphila* (*Verrucomicrobiota*) and an increased abundance of *Proteobacteria* have been documented [23,26]. These microbial alterations contribute to impaired intestinal homeostasis, including increased epithelial permeability and gut barrier dysfunction [27]. Consequently, such dysbiosis promotes systemic metabolic disturbances, including chronic low-grade inflammation and insulin resistance.

To counteract these obesity-related microbial shifts, the dietary integration of legumes such as soy and lupin has emerged as a promising strategy. Both seeds contain bioactive compounds that modulate the composition and diversity of the gut microbiome, eliciting systemic beneficial effects [28,29]. These legumes selectively promote the proliferation of commensal taxa, including *Bifidobacterium*, *Faecalibacterium*, and *Lactobacillus* species, while concurrently suppressing pathogenic populations [30,31]. Furthermore, in vivo studies have demonstrated that the administration of soy or lupin proteins promotes weight loss and reduces adiposity in murine models of high-fat diet-induced obesity [32,33]. Specifically, soy protein has been shown to enhance the secretion of intestinal glucagon-like peptide-1 (GLP-1) and increase the production of secondary bile acids, a process closely linked to the enrichment of microbiota involved in bile acid biotransformation [33]. Complementarily, lupin protein administration restores key commensal bacteria that facilitate the attenuation of insulin resistance and dyslipidemia [32].

Given that dietary interventions are a cornerstone for managing metabolic disorders, soy and lupin-based nutritional formulations emerge as a promising strategy to mitigate the disease burden associated with obesity from early life stages. Therefore, in the present study, we aimed to evaluate the clinical effects of two supplements formulated either with soy or lupin protein in Mexican male adolescents with overweight and obesity. We evaluated multiple biochemical, anthropometric, and metabolic parameters, including adipocytokines and incretin circulating levels, as well as the profile of the gut microbiome before and after a five-week intervention period.

## 2. Materials and Methods

### 2.1. Study Design

A randomized, double-blind study was designed to evaluate the effects of the protein-enriched lupin (LB) and soy beverages (SB). To minimize the influence of dietary heterogeneity on biochemical, clinical, and gut microbiota parameters, this study was conducted among adolescents at a boarding school in Guadalajara, Mexico. The institution provides care exclusively for male adolescents; therefore, only males were eligible for recruitment in the present study. During the study period, the adolescents remained at the institution from Monday through Saturday, while Sundays were spent with their families before returning to the facility on Sunday evening. A cohort of 92 male adolescents (aged 12–16 years) was initially recruited as potential participants.

The adolescents and their parents or guardians received a detailed explanation of the study before accepting their participation and signing their informed assent and consent, respectively. The study protocol was in accordance with the Declaration of Helsinki and was approved by the Bioethics Committees at both the Hospital Civil de Guadalajara (012/14 HCJM/2014) and the Centro Universitario de Ciencias de la Salud, Universidad de Guadalajara (number CI-01214). Comprehensive baseline data were collected for all participants, including demographics, lifestyle habits, and detailed clinical and family medical histories.

### 2.2. Anthropometry

To identify eligible individuals, anthropometric measurements were performed by certified nutritionists in accordance with standardized clinical protocols. The individuals’ measurements were recorded without shoes and wearing light clothing at 7:00 h at the beginning of the trial. Body weight, height, and waist circumference were measured using a Seca 700 mechanical scale, a Seca 217 stadiometer (Hamburg, Germany) and a Lufkin W606PM metal tape (Sparks, MD, USA), respectively. The waist circumference was measured to the nearest millimeter above the uppermost lateral border of the ilium at the end of a normal expiration cycle. Total body fat percentage was measured with the BIA (Bioimpedance Analysis) device Tanita^®^ BC-532 (Tokyo, Japan). Body mass index (BMI), height-for-age z-score, and BMI-for-age z-score were calculated using the WHO Anthro plus software for personal computers (version 1.0.4 World Health Organization (WHO), Geneva, Switzerland).

### 2.3. Selection of Participants

**Figure 1:**
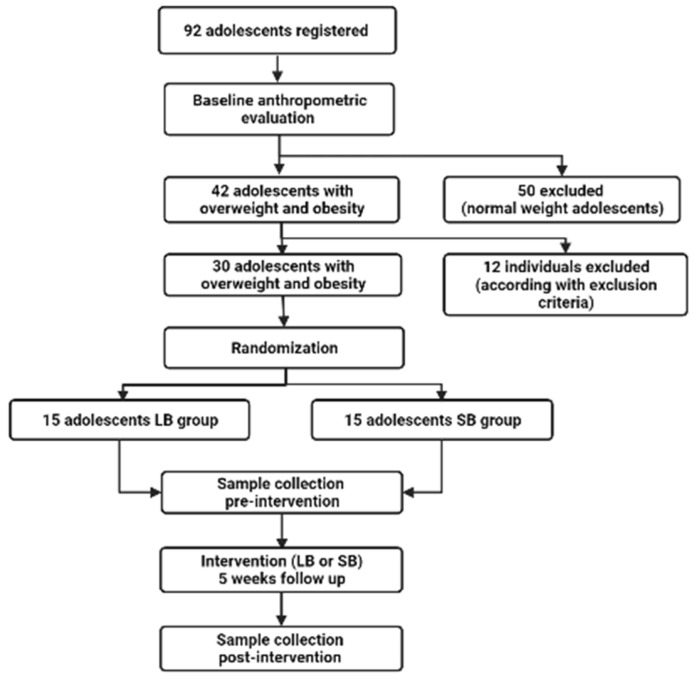
Selection of participants in this study.

Participants were classified according to BMI-for-age criteria; consequently, 42 adolescents met the criteria for overweight (*n* = 23) or obesity (*n* = 19) and were enrolled in the study. Of these 42 subjects, 12 were excluded for not meeting the eligibility criteria; thus, a final sample of 30 individuals was reached. These participants (overweight, *n* = 15; obesity, *n* = 15) were then randomly allocated into the two intervention groups, as shown in the flow diagram (Figure 1).

The following exclusion criteria were considered: previous diagnosis of endocrine, cardiac, renal or chronic abnormalities, and any other pathology associated with obesity or diabetes mellitus; weight loss in the last six months, as well as treatment with any medication that could affect the cardiovascular or metabolic function. None of the participants received anti-inflammatory drugs or medications that influence metabolism, blood pressure, or sympathetic nervous system activity.

### 2.4. Nutritional Assessment

At the beginning of the study, a baseline nutritional assessment was performed using a 24 h dietary recall to estimate habitual energy and nutrient consumption. These records, which detailed all food and beverage intake, were reviewed by a certified nutritionist to verify portion sizes, cooking methods, ingredients, and consumption timing. Subsequently, dietary data were processed using a customized database based on the Sistema Mexicano de Alimentos Equivalentes (SMAE) [34] for the precise quantification of macronutrient distribution and total caloric intake. Participants resided in the boarding school where a common menu was provided to all adolescents. Breakfast, lunch, dinner, and snacks were prepared centrally by the institution and offered equally to all adolescents, without individualized nutritional prescription or weight-management dietary programs.

The study did not include dietary counseling or modification to the habitual institutional menu, and participants were encouraged to maintain their usual eating patterns throughout the intervention period. Therefore, the supplementation with LB or SB represented the only planned nutritional modification introduced during the study.

### 2.5. Interventional Phase: Beverage Supplementation

The selected adolescents (overweight, *n* = 15; obesity, *n* = 15) were randomly assigned to one of two double-blind intervention groups, ensuring an equal distribution of nutritional status across both arms. Group composition was compared using Fisher’s exact test, which confirmed no significant differences in the proportion of overweight and obese individuals between groups (*p* > 0.05; Table S1). Subsequently, the effects of supplementation with either the SB or LB were evaluated over a five-week intervention period. To minimize potential dietary confounding, no additional dietary interventions were implemented during the supplementation period, and the institutional meal plan remained unchanged throughout the study.

The SB group received a beverage with soy protein (10 g), and the second one a beverage with lupin protein (10 g). A portion, 25 g, of beverage’s powder form (containing 10 g of the respective protein), was dissolved in 300 mL of cold water. Both beverages were freshly prepared every morning (10:00 h) and given daily at snack time (10:30 h) to the participants for 5 weeks. In a preliminary test, three flavors of the beverages were evaluated (vanilla, strawberry, and chocolate). The participants reported higher palatability to the chocolate flavor, which was selected for the intervention.

The functional beverages were formulated using a standardized matrix designed to deliver specific concentrations of isolated proteins. Each formulation was based on either soy (SB) or lupin protein isolate (LB), balanced with a controlled proportion of carbohydrates, fats, and dietary fiber. This approach ensured that the physiological effects observed could be attributed to the specific protein source and its associated fiber content, while maintaining a consistent caloric density across both treatment groups (Table 1).

### 2.6. Biochemical Parameters, Adipocytokines, and Hormones

Biochemical, anthropometric parameters, and adipocytokine levels were evaluated in adolescents with obesity and overweight before and after the five-week intervention period. Blood samples, taken from each participant after 12 h of fasting, were collected in tubes without anticoagulant for all determinations. Fasting serum glucose, total cholesterol, triglycerides (TG), high-density lipoprotein cholesterol (HDL-c), aspartate aminotransferase (AST), and alanine aminotransferase (ALT) levels were quantified in a semi-quantitative spectrophotometer (BTS-350; BioSystems, Barcelona, Spain) using analytical grade reagents purchased from BioSystems (BioSystems, Barcelona, Spain). Low-density lipoprotein cholesterol (LDL-c) was calculated using Friedewald’s formula: [LDL-c] = [Total chol] − [HDL-c] − ([TG]/5).

We also quantified total serum plasminogen activator inhibitor-1 (PAI-1), visfatin, resistin, glucose-dependent insulinotropic peptide (GIP), insulin, C peptide, and glucagon following the manufacturer’s instructions for the Bio-Plex pro multiplex system kit (BIO-RAD, Hercules, CA, USA) and analyzed in a Magpix system (Luminex, Austin, TX, USA). Enzyme-linked immunosorbent assays (ELISA) were used to measure adiponectin, human total adiponectin/Acrp30, human leptin, human/canine/porcine insulin and human C-peptide (R&D Systems, Minneapolis, MN, USA). The HOMA2 index was calculated using the software developed by Oxford University version 2.2.4, using fasting glucose and C-peptide serum levels [35].

### 2.7. Gut Microbiome Analysis

Fecal samples were collected both prior to the beginning of the study and after five weeks of administration of the intervention. Initial samples were collected one day prior to beverage administration, while the final samples were collected on the next day following the completion of the five-week period. All samples were freshly collected in sterile tubes and promptly frozen in dry ice, then stored at −80 °C until analysis.

The fecal samples underwent DNA extraction using the Quick-DNA Fecal/Soil Kit (Zymo Research, Irvine, CA, USA) following the manufacturer`s instructions. Analysis of the gut microbiome focused on five principal phyla (*Bacillota*, *Bacteroidota*, *Verrucomicrobiota*, *Actinomycetota*, and *Pseudomonadota*), as well as the presence of *butyryl-CoA transferase* (BUT) gene-positive bacteria and the *Akkermansia muciniphila* species. Total bacteria quantification was achieved by amplifying the V3 to V4 region of the 16S rDNA gene, with primer sequences and annealing temperatures shown in detail (Table 2). Real-time PCR quantification was conducted using Maxima SYBR Green Master Mix (Thermo Fisher Scientific, #K0251, Waltham, MA, USA) and the StepOne Thermocycler (Applied Biosystem, Waltham, MA, USA).

The PCR conditions for amplification of the 16S rDNA gene included an initial denaturation at 95 °C for 10 min, followed by 25 cycles of 95 °C for 30 s, 60 °C for 50 s, and 72 °C for 40 s. For the gut microbiome phyla, BUT-gene and *A. muciniphila*, reactions began with an initial denaturation at 95 °C for 10 min, followed by 40 cycles of 95 °C for 30 s, annealing at the temperature specified in Table 2, and an extension stage of 72 °C for 40 s.

All primer sequences were analyzed and corroborated using the BLAST program (available at: https://blast.ncbi.nlm.nih.gov/Blast.cgi, accessed on 30 April 2026) from the National Center for Biotechnology Information to ensure accurate alignment with the target sequences. Relative quantification of PCR products was determined for the 16S rDNA using the 2^ΔCt^ method, whereas for each phylum and species, the 2^ΔΔCt^ method was used. The 16S rDNA gene served as the reference. Additionally, a melting curve analysis was performed for each analyzed gene to confirm the amplicon’s specificity.

### 2.8. Statistical Analysis

Data are represented as the mean ± standard deviation (SD) or mean ± standard error of the mean (SEM), as indicated in each figure. Student’s *t*-test was used to establish differences between groups. A two-way ANOVA test was conducted to analyze the gut microbiota abundance and the differences between the pre- and post-supplementation within each group. Tukey’s multiple comparison test was applied for post hoc analyses. Pearson’s correlation coefficients were calculated to evaluate associations between gut microbiota composition and biochemical, anthropometric, and metabolic parameters at baseline and post-treatment in both study groups. Statistically significant changes were considered when *p*-values were ≤0.05. The analyses were computed with IBM SPSS Statistics v. 20.0 software for Windows (Armonk, NY, USA) and GraphPad Prism v.10 software (Boston, MA, USA).

## 3. Results

### 3.1. Nutritional and Anthropometric Evaluation of Study Subjects

The baseline evaluation of nutritional and anthropometric parameters showed that average daily energy intake and physical activity levels were not different between LB and SB study subjects. Macronutrient distribution and overall dietary composition were similar, exhibiting no significant differences in protein, fat, carbohydrate, dietary fiber, water consumption, or added sugar intake between the SB and LB groups. Likewise, no significant differences were found in demographic or anthropometric characteristics of the groups. Moreover, the quality of the diet, assessed using the Healthy Eating Index (HEI)-2020, was comparable between the SB and LB groups, showing similar total HEI or component scores (Table 3).

Anthropometric parameters were evaluated at baseline and after the five-week intervention period. We observed a statistically significant increase in the weight of the individuals from the SB group compared to their pre-intervention values. In contrast, a slight weight increase in the LB group was found (not significant). Similarly, the height of the individuals exhibited a significant increase in both intervention groups. These anthropometric changes, however, correlated with the age of the subjects and were expected to occur during adolescence. Considering this, we decided to complement our data by analyzing the height-for-age growth indicator and the BMI-for-age growth indicator, as a more reliable evaluation for changes according to the age of the study subjects. These parameters did not show significant differences between pre- and post-intervention values.

On the other hand, the assessment of the body fat percentage did not reveal any significant differences after the intervention. Similarly, the evaluation of the waist circumference of the individuals showed no differences. The results of the anthropometric parameters are summarized in Table 4.

### 3.2. Biochemical Parameters

Previous studies have suggested a positive influence of lupin and soy proteins on metabolic parameters. Thus, we were particularly interested in assessing whether metabolism-related blood serum analytes were modified at the end of the interventions. Notably, we observed a subtle decrease in the fasting blood glucose levels of both SB and LB groups (−6.52 and −5.57 mg/dL, respectively) compared to their initial values. Closely related to the blood glucose levels, we also determined the circulating insulin and C-peptide levels. C-peptide levels were significantly decreased after the intervention in both groups. In accordance with the changes in serum glucose and C-peptide levels, the evaluation of the HOMA2-IR revealed a significant reduction in this parameter by −18.8% and −15.9% after the SB and LB interventions, respectively. Glucagon, an insulin-counterpart in blood glucose regulation, showed a statistically significant decrease by the intervention in the LB group. Moreover, the total cholesterol (−11.4%) and triglycerides (−18.5%) were significantly decreased in the SB group (*p* < 0.05). Although significant decreases were detected in the urea and creatinine concentrations, these changes are within the normal value ranges for these analytes. A comparison of baseline and changes after treatment of all parameters is presented in Supplementary Materials (Tables S2 and S3).

### 3.3. Metabolic Parameters

In addition to biochemical evaluation, we decided to investigate whether the intervention with the legume proteins had any effects on additional circulating hormones relevant for metabolic homeostasis. To address this, we evaluated parameters related to glucose metabolism, appetite, as well as cytokines produced and released to circulation by the adipose tissue.

**Figure 2:**
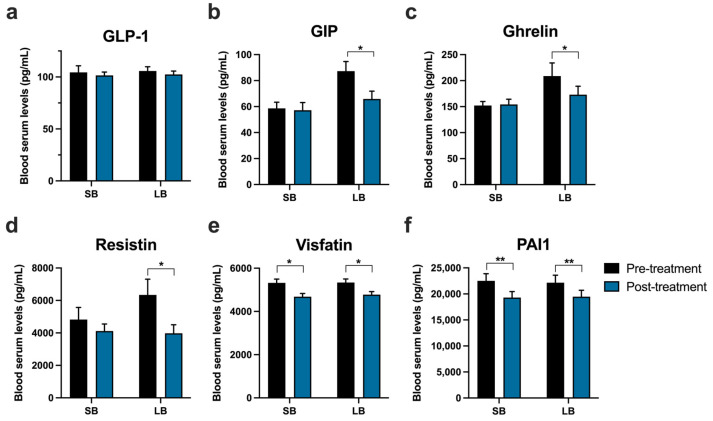
Incretins GIP and GLP-1, ghrelin, plasminogen activator inhibitor-1 (PAI1), resistin and visfatin levels before and after supplementation with SB or LB. The levels of (**a**) GLP-1, (**b**) GIP, (**c**) ghrelin, (**d**) resistin, (**e**) visfatin, and (**f**) PAI-1 were determined in the blood serum of patients before and after a 5-week intervention with SB and LB. * *p* ≤ 0.05, ** *p* ≤ 0.01 statistical differences between pre- and post-supplementation. SB, *n* = 15; LB *n* = 15. Data are represented as mean + SEM.

Whereas the blood serum levels of the incretin GLP-1 did not exhibit any difference after the 5-week intervention, either with SB or LB, GIP was significantly decreased in LB-supplemented subjects (Figure 2a,b). Similarly, ghrelin levels were significantly lower in the LB group by −16% (Figure 2c). However, baseline levels of GIP and ghrelin were significantly higher in the LB group compared to the SB group (*p* = 0.004 and *p* = 0.042, respectively).

We also compared the effect of both supplements on the concentration of adipocytokines. We observed that in both the SB and LB groups, the concentration of leptin tended to decrease (Table 2). The plasminogen activator inhibitor-1 (PAI-1), on the other hand, was −12% and −14% lower after the LB and SB consumption, respectively. The concentration of resistin was also reduced in both groups, but only significant in the LB group. Baseline levels of resistin were not significantly different between SB and LB groups. Visfatin was significantly reduced in both groups by −12% and −10% for soy and lupin groups, respectively (Figure 2).

### 3.4. Gut Microbiome Composition

**Figure 3:**
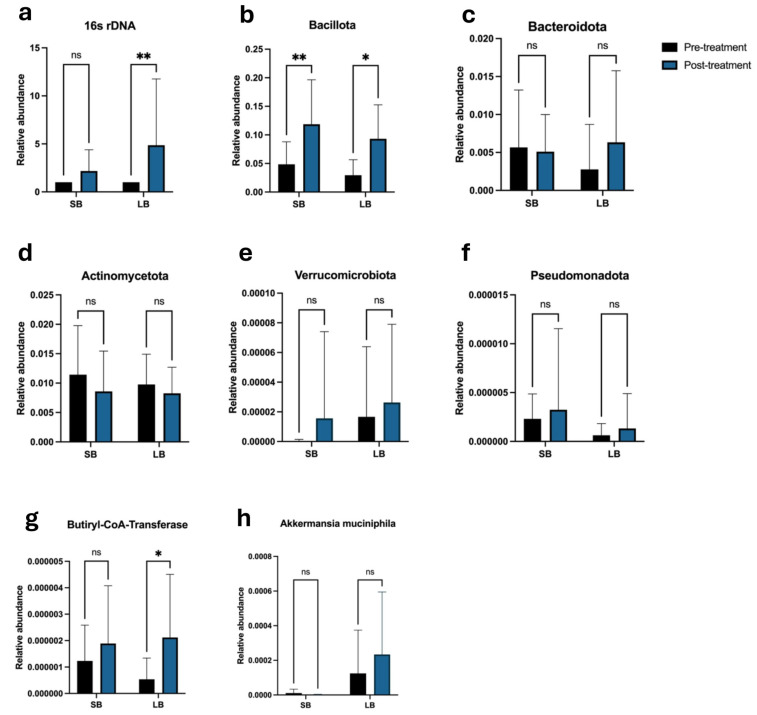
Gut microbiome composition of children with overweight and obesity before and after the administration of SB and LB. Relative quantification of gut microbiome was determined using 2^ΔΔ^Ct analysis, comparing the 16S rDNA as a constitutive gene with each specific determination (Phylum and BUT genes). The panels display relative abundances of (**a**) 16S rDNA, (**b**) *Bacillota* phylum, (**c**) *Bacteroidota* phylum, (**d**) *Actinomycetota* phylum, (**e**) *Verrucomicrobiota* phylum, (**f**) *Pseudomonadota* phylum, (**g**) BUT gene-positive bacteria, and (**h**) *Akkermansia muciniphila*. Pre- and post-treatment values are represented by the black and blue bars, respectively. Differences were analyzed using Two-way ANOVA with treatment and time as fixed factors, followed by Tukey’s multiple comparison test. ns, non significant; * *p* ≤ 0.05, ** *p* ≤ 0.01 indicate significant differences between pre- and post-intervention values. SB, *n* = 15; LB, *n* = 14.

To further investigate the effects of SB and LB supplementation on gut microbiota composition, changes in total bacterial abundance and specific microbial groups were evaluated by semiquantitative real-time PCR. Total bacterial abundance, assessed through amplification of the 16S rDNA gene, showed a significant increase after the intervention in both groups. Notably, adolescents receiving the LB exhibited a marked fourfold increase in total bacterial abundance after supplementation (Figure 3a).

Analysis at the phylum level revealed a significant increase in the relative abundance of *Bacillota* in both intervention groups, with approximately twofold and threefold increases in the SB and LB groups, respectively (Figure 3b). The *Bacteroidota* phylum showed a tendency to increase in the LB group, whereas no change was observed in the SB group (Figure 3c). On the other hand, *Actinomycetota* abundance tended to decrease following supplementation with both the SB and the LB (Figure 3d). Minor increases were observed in the abundance of *Verrucomicrobiota* and *Pseudomonadota* phyla; however, these changes did not reach statistical significance (Figure 3e and Figure 3f, respectively).

Within the *Verrucomicrobiota* phylum, the abundance of *Akkermansia muciniphila* was specifically quantified due to its established role in mucus layer maintenance and metabolic health. No significant differences were observed in *A. muciniphila* abundance either on the SB or LB groups (Figure 3h).

To explore bacterial function potentially related to short-chain fatty acid production, the abundance of bacteria harboring the *Butyryl-CoA: acetate CoA transferase* (BUT) gene was evaluated. A significant fourfold increase in the abundance of this gene was detected following LB supplementation (Figure 3g). In the SB group, the abundance of the BUT gene-positive bacteria increased approximately 46%; however, this change did not reach statistical significance.

### 3.5. Associations Between Gut Microbiota Composition and Clinical-Metabolic Parameters

**Figure 4:**
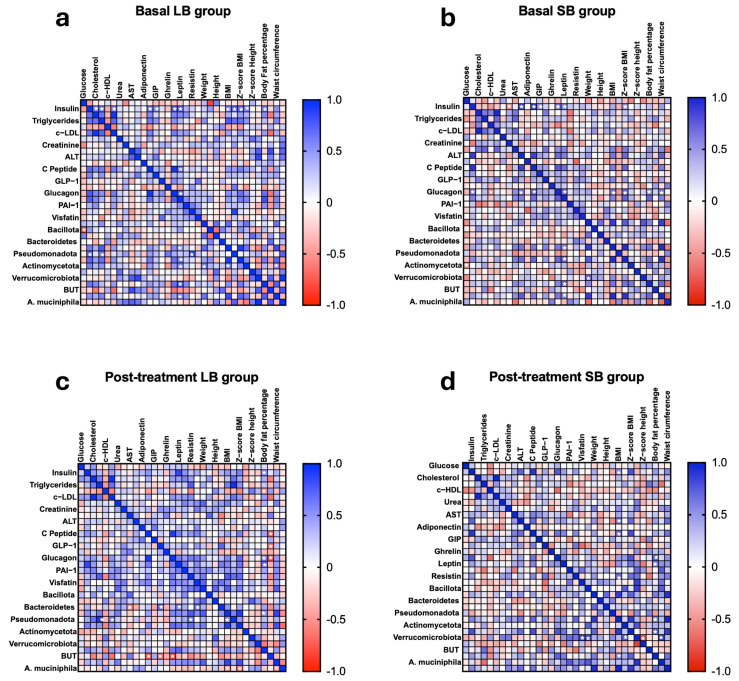
Associations between gut microbiota composition and clinical-metabolic parameters in children with overweight and obesity. Pearson correlation heatmaps showing the relationships between gut microbiota taxa and clinical, anthropometric, and metabolic variables. White asterisks (*) indicate statistically significant correlations (* *p* < 0.05). Blue colors represent positive correlations, whereas red colors represent negative correlations. (**a**) Baseline LB group; (**b**) baseline SB group; (**c**) post-treatment LB group; and (**d**) post-treatment SB group. SB, *n* = 15; LB, *n* = 14. ALT, alanine aminotransferase; AST, aspartate aminotransferase; BMI, body mass index; BUT: *Butiryl-CoA transferase* gene; GIP, glucose-dependent insulinotropic polypeptide; GLP-1, glucagon-like peptide-1; c-HDL, cholesterol in high-density lipoproteins; c-LDL, cholesterol in low-density lipoproteins; PAI-1, plasminogen activator inhibitor-1.

To investigate the associations between gut microbiota composition and biochemical, anthropometric, and metabolic parameters pre- and post-treatment in both groups, Pearson correlation analyses were performed (Figure 4).

At baseline, different correlation patterns were observed between microbial taxa and metabolic markers in both intervention groups (Figure 4a,b). In the LB group, *Pseudomonadota* abundance was positively associated with insulin, triglycerides, and resistin, whereas *Actinomycetota* showed positive correlations with adiposity-related parameters, including BMI, waist circumference, body weight, and leptin levels. In contrast, *Bacillota* abundance was negatively correlated with glucose concentrations (Figure 4a). Similarly, in the SB group, *Actinomycetota* abundance was inversely associated with glucose levels, while *Verrucomicrobiota* abundance was positively correlated with body weight. In both groups, adiposity-related biomarkers, particularly leptin and insulin, exhibited strong positive associations with anthropometric and metabolic variables (Figure 4a,b).

Following the intervention, microbiota-metabolic associations became more pronounced in the LB group. Notably, *Akkermansia muciniphila* abundance was inversely correlated with BMI, BMI z-score, and waist circumference, suggesting a potential association with improved metabolic status. Likewise, the relative abundance of BUT gene-positive bacteria was negatively associated with C-peptide, GLP-1, and glucagon levels. Conversely, *Pseudomonadota* abundance remained positively associated with unfavorable metabolic markers, including triglycerides, LDL cholesterol, resistin, and BMI. In addition, *Bacteroidota* abundance was positively correlated with GLP-1, leptin, and visfatin and negatively correlated with AST levels (Figure 4c).

In the post-treatment of the SB group, *Verrucomicrobiota* abundance was positively associated with visfatin levels, whereas positive correlations were observed among several microbial taxa, including *Bacillota, Actinomycetota*, and *Akkermansia muciniphila*. Furthermore, body fat percentage and BMI remained strongly associated with anthropometric, hormonal, and inflammatory markers (Figure 4d).

Overall, these findings suggest that both interventions modulated microbiota-host interactions. The lupin-based beverage showed stronger associations between beneficial microbial taxa, particularly *Akkermansia muciniphila* and BUT gene-positive bacteria, with improved metabolic outcomes.

## 4. Discussion

In the present study, we evaluated the effect of two plant protein-based functional beverages (soy and lupin) that were supplemented daily during five weeks on the diet of male adolescents with overweight or obesity. The observed effect of both functional beverages was similar for more than one of the studied variables, showing a reduction in cardiometabolic risk indicators. To the best of our knowledge, this is the first interventional study exploring the effects of lupin- and soy-protein-based beverages in children with overweight and obesity.

The hypoglycemic effect of soy and lupin proteins has been extensively studied in experimental models [20,42,43,44] as well as in some clinical trials with adult populations [19,45,46]. Our data showed a reduction in blood glucose and C-peptide levels that led to a significant decrease in the HOMA2-IR values of LB and SB groups. Similarly, a previous randomized clinical trial has shown that soy consumption reduces blood glucose, C-peptide, and HOMA-IR values in postmenopausal women when consumed for a period of three weeks [11]. Moreover, the hypoglycemic effect of lupin protein has also been reported in clinical and animal studies following acute [19] and chronic administration of lupin [47], respectively. Remarkably, our findings indicate an increase in insulin sensitivity (HOMA2-%S) without changes in β-cell function (HOMA2-%B), together with reduced basal C-peptide and glucagon levels. These changes suggest an improved metabolic efficiency characterized by a potential reduction in insulin demand and restored insulin-mediated suppression of glucagon secretion.

A previous study suggested an inhibitory effect of soy and lupin-derived peptides on the activity of human DPP-IV [48]. These could lead to higher levels of incretin hormones GIP and GLP-1, which can suppress glucagon secretion [49]; however, we observed a reduction in GIP levels only in the LB group. This scenario more likely reflects systemic metabolic adaptations, implicating an enhanced insulin sensitivity in pancreatic α-cells that might restore the insulin-mediated suppression of glucagon secretion, contributing to reduced basal glucagon levels.

Ghrelin, an orexigenic intestinal peptide [50] produced by gastric oxyntic cells in mice and P/D1 cells in humans, plays a key role in stimulating appetite at the hypothalamic level, among other physiological processes [51,52]. Previous studies have shown a negative correlation between ghrelin levels and body weight, suggesting that obesity is associated with lower ghrelin levels. In fact, total ghrelin levels are influenced by the individual’s nutritional condition. Ghrelin tends to increase during fasting, while its levels decrease after food intake [53]. A meta-analysis by Wang et al. concluded that ghrelin levels during fasting are lower in individuals with obesity, while postprandial levels remain similar to those of healthy subjects [54]. In accordance with our findings, a clinical study demonstrated that administering a bean extract to healthy subjects suppressed plasma ghrelin levels for up to three hours after consumption, prolonging satiety and reducing the desire to eat [55]. Similarly, Lee et al. evaluated the effect of consuming bread supplemented with lupin flour on ghrelin concentration in healthy adults [17]. Like beans, lupin flour maintained lower ghrelin levels for a longer period compared to wheat flour bread. Accordingly, we observed that in the SB group, ghrelin remained unchanged, whereas in the LB group its levels were reduced.

We decided to also assess inflammation-related molecules, since some reports have suggested anti-inflammatory effects of legumes like lupin and soy [56]. PAI-1, a molecule that can be produced by adipocytes and macrophages, among others, has been associated with cardiovascular disease [57]. Therefore, PAI-1 levels are increased in obesity and metabolic syndrome patients [58]. Here, the supplementation of both lupin and soy proteins led to decreased PAI-1 serum concentration, in parallel with a reduction in visfatin and resistin levels. Serum visfatin levels are higher in children with obesity than in normal-weight children. Moreover, visfatin levels are significantly elevated when obese individuals exhibit metabolic syndrome compared to those with obesity [59,60]. In addition, resistin plays a crucial role in the chronic, low-grade inflammation associated with obesity by activating the nuclear transcription factor-kappa B, promoting multiple inflammatory pathways that contribute to endothelial dysfunction, vascular smooth muscle cell proliferation, hypertension, and coronary artery disease [61,62]. The concomitant reduction in and regulation of these adipocytokines (PAI-1, resistin, and visfatin) through the administration of SB and LB suggests a modulatory effect on inflammatory mediators, which may contribute to reducing systemic inflammation and mitigating obesity-related metabolic complications. Notably, a decrease in low-grade obesity-related inflammation aligns well with the improvements in insulin sensitivity and insulin demand, previously described. Altogether, improvements in these parameters, together with enhanced systemic insulin sensitivity observed following the LB and SB interventions, suggest a healthier adipose tissue metabolic status. Nevertheless, additional measurements, including fasting free fatty acid concentrations, should be addressed in future studies to confirm improved adipose tissue insulin sensitivity.

On the other hand, the gut microbiota has been proposed as a promising and modifiable therapeutic target to improve health through the administration of bioactive compounds in various diseases, including obesity [63]. The gut microbiota constitutes a complex ecosystem of microorganisms residing in the gastrointestinal tract, with bacteria representing the predominant population. This microbial community is mainly organized into five major phyla: *Bacillota, Bacteroidota*, *Verrucomicrobiota*, *Actinomycetota* and *Pseudomonadota* [64]. The phylogenetic distribution of gut microbiota is influenced by several factors, including host genetics, health status, and environmental exposure. Notably, the dietary habits play a critical role in shaping microbial diversity and phylogenetic composition, thereby influencing host metabolism [63,64].

In obesity, both bacterial concentration and microbial diversity are known to be reduced. In this context, the use of legumes as sources of bioactive compounds has gained increasing attention as a strategy to improve gut microbial communities. Soy and lupin contain a variety of bioactive compounds beneficial for the gut microbiota, particularly due to their high oligosaccharide content [65,66]. Consequently, these legumes have been studied for their potential to modulate gut microbiota composition [66,67].

In our study, the administration of soy and lupin protein beverages improved the relative abundance of the 16S rDNA gene, particularly in the LB group, suggesting an enhancement in microbial richness. One of the main phylogenetic changes observed was an increase in the *Bacillota* phylum in both groups. This increase could be associated with a higher abundance of butyrate-producing bacteria. Consistently, our results showed that LB supplementation increased the abundance of bacteria harboring the BUT-gene. Key butyrate-producing bacteria, such as *Eubacterium rectale* and *Roseburia intestinalis,* play a well-recognized role in butyrate production, which is often reduced in individuals with obesity [68]. Accordingly, the reduction in butyrate-producing bacteria has been linked to weight gain, visceral fat accumulation, and insulin resistance [69,70]. Supporting the metabolic relevance of BUT-positive bacteria, our correlation analysis revealed a negative association between their abundance and the post-intervention concentration of C-peptide, GLP-1, and glucagon. Although these associations do not establish causality, they may partially explain the beneficial metabolic shifts observed. However, the concurrent improvements in microbiota composition and metabolic outcomes do not allow causal inferences. The increased intake of legume-derived proteins and dietary fiber may have promoted the growth of beneficial bacteria, thereby contributing to improved metabolic health. Conversely, the improvement in metabolic status may have modified the intestinal environment in a manner that further supported microbiota remodeling. Thus, the relationship between gut microbiota and metabolic health is likely bidirectional, with dietary intervention acting as a common driver of both responses. This interpretation is supported by previous studies reporting that supplementation with soy, lupin seeds, or their isolated oligosaccharides enhances microbial richness and promotes the growth of butyrate-producing bacteria, improving the intestinal homeostasis and metabolic health [30,71,72,73].

In addition to the aforementioned taxa, this research also monitored the response of the phylum *Pseudomonadota*. Our correlation analysis highlighted *Pseudomonadota* as a persistent marker of metabolic dysfunction. At baseline, this phylum was positively correlated with insulin, triglycerides, and resistin. Following the intervention in the LB group, it maintained positive associations with triglycerides, LDL cholesterol, resistin, BMI, and other metabolic risk factors. These results are consistent with previous reports identifying *Pseudomonadota* as a microbial hallmark of obesity and metabolic disorders [74,75,76]. Mechanistically, this phylum encompasses several Gram-negative bacteria that produce lipopolysaccharides (LPS), which are known to drive low-grade systemic inflammation, insulin resistance, and lipid metabolism disturbances [77,78]. Therefore, the positive correlations with adiposity and inflammatory markers reinforce the role of *Pseudomonadota* as a consequence of a metabolically unfavorable gut ecosystem in children with overweight and obesity. However, due to the cross-sectional and correlational nature of these data, these findings may indicate ongoing microbiota-host crosstalk rather than causal associations.

To gain a more comprehensive overview of the taxonomic shifts, our evaluation also encompasses the analysis of the phylum *Verrucomicrobiota* and *Akkermansia muciniphila*. Our results showed that both LB and SB supplementation were associated with an increase in the abundance of this phylum, while LB specifically promoted a higher relative abundance of *A. muciniphila*. This bacterium is typically reduced in individuals with overweight and obesity, and has been linked to impaired intestinal barrier function [79]. Although these changes did not reach statistical significance, a notable upward trend was observed, particularly in the LB group, where *A. muciniphila* abundance was doubled following supplementation. This tendency may suggest a biologically relevant effect that could become more evident with longer intervention periods or larger sample sizes. Additionally, post-treatment correlation analysis in the LB group revealed an inverse association between *A. muciniphila* abundance and anthropometric markers, including BMI, BMI Z-score, and waist circumference. The relevance of this observation aligns with previous reports indicating that higher levels of administration of *A. muciniphila* are associated with reductions in body weight, fat mass, and improvements in metabolic parameters, whereas lower abundance has been linked to adverse outcomes. Therefore, even in the absence of statistical significance in the abundance of *A. muciphila* after the beverages, the observed trend may represent an early indicator of microbiota modulation and highlights the need for further studies to evaluate the long-term impact of legume-based interventions, such as soy and/or lupin, on beneficial microbial taxa and their relationship with clinical parameters associated with overweight and obesity [32].

Taken together, our results suggest a role of legume proteins in improving the metabolic status and its potential capacity to modulate the bacterial community in western Mexican adolescents with either overweight or obesity. This study also highlights the relevance of supplementing diets with functional beverages that positively impact health, especially in anthropometric, biochemical, metabolic, and intestinal health indicators.

Limitations of the study: Several limitations should be acknowledged in the present study. We compared two active plant-protein supplements and did not include a non-supplemented control group; therefore, the results should be interpreted relative to baseline and between interventions. In addition, only male adolescents were included, limiting the extrapolation of the findings to females. Moreover, since obesity has been linked to altered estrogen levels and soy isoflavones may interact with estrogen-related pathways, future studies are required to identify whether sex hormone levels are influenced by the treatment. On the other hand, dietary intake was assessed only at baseline using a 24 h recall, and physical activity, appetite, and satiety were not monitored during the intervention; thus, we cannot rule out that changes in energy intake, eating behavior, or activity levels may have contributed to the observed responses. Overweight and obesity were also analyzed together because of the sample size, which may have masked differences related the response to the treatments mediated by adiposity status. Finally, gut microbiota analysis was based on targeted RT-qPCR, limiting the detection of broader taxonomic and functional changes. Future studies employing genome sequencing technologies will help to identify additional microbiota populations that could play a role in the beneficial metabolic effects found in our study.

## 5. Conclusions

In conclusion, the present study shows the potential clinical benefits of including lupin or soy proteins in the management of obesity and its related metabolic disorders in male adolescents. These findings also suggest that the incorporation of legume proteins into the diet offers beneficial effects on metabolic health by enriching and influencing gut microbiota composition. Based on the beneficial effects found to correlate with these two plant-based beverages, they could be considered as complementary management alternatives for overweight and obesity in male adolescent populations. Similar studies in female adolescents are needed to clarify whether these metabolic and microbiota-related effects are sex-independent.

## Figures and Tables

**Table 1:** Macronutrient composition and nutritional profile of the lupin and soy protein-based beverage powders used in the intervention.

Ingredients	Lupin (25 g)	Soy (25 g)
Lupin protein isolate (g)	10	−
Soy protein isolate (g)	−	10
Total fat (g)	0.36 g	0.36
Total carbohydrate (g)	14.97	14.97
Fiber (g)	1.87	1.87
Energy content (nutrient values)		
Protein (kcal)	40	40
Fat (Kcal)	3	3
Carbohydrate (Kcal)	60	60
Total Energy (Kcal)	103	103

The symbol “−” indicates parameters not applicable for the corresponding category.

**Table 2:** Sequences and annealing temperature of the primers used for gut microbiome analysis.

Primer	Sequence (5′ -> 3′)	Amplicon Size (bp)	Annealing Temperature (°C)	Reference
16S rDNA	27F: AGAGTTTGATCMTGGCTCAG 519R: GWATTACCGCGGCKGCTC	405 ± 61	60	[36]
*Bacillota*	F: GGAGYATGTGGTTTAATTCGAAGCA R: AGCTGACGACAACCATGCAC	126	67	[37]
*Bacteroidota*	F: GGAGYATGTGGTTTAATTCGAAGCA R: AGCTGACGACAACCATGCAG	126	65	[37]
*Actinomycetota*	F: TGTAGCGGTGGAATGCGC R: AATTAAGCCACATGCTCCGCT	280	65	[38]
*Verrucomicrobiota*	F: TCAKGTCAGTATGGCCCTTAT R: CAGTTTTYAGGATTTCCTCCGCC	100	62	[38]
*Pseudomonadota*	F: TCGTCAGCTCGTYGTGA R: CGTAAGGGCCATGATG	154	62	[39]
BUT	F: GCIGAICATTTCACITGGAAYWSITGGCAYATG R: CCTGCCTTTGCAATRTCIACRAANGC	530	53	[40]
*Akkermansia muciniphila*	F: CAGCACGTGAAGGTGGGGAC R: CCTTGCGGTTGGCTTCAGAT	343	65	[41]

Primer orientation: F, forward; R, reverse. Degenerate primers: I = Inosine; K = G or T; M = A or C; N = A, G, C or T; R = A or G; S = G or C; Y = C or T; W = A or T.

**Table 3:** Baseline nutritional parameters and physical activity evaluation.

Value	SB Group	LB Group	*p* Value
Energy (kcal/day)	1988.0 ± 389.8	1944.0 ± 422.2	0.99
Carbohydrates (g/day)	265.0 ± 65.57	270.0 ± 87.56	0.88
Protein (g/day)	85.3 ± 19.9	78.4 ± 21.4	0.51
Lipids (g/day)	64.6 ± 16.2	60.9 ± 13.4	0.64
Sugar (g/day)	69.2 ± 36.5	103.5 ± 43.5	0.09
Carbohydrate (%)	53.0 ± 6.0	55.0 ± 8.7	0.68
Sugar (%)	16.0 ± 8.9	21.0 ± 7.6	0.20
Protein (%)	17.0 ± 3.2	16.0 ± 2.8	0.31
Lipid (%)	29.0 ± 6.3	29.0 ± 8.9	0.93
Water (L/day)	1.7 ± 1	1.8 ± 0.7	0.93
Fiber (g/day)	16.9 ± 8.6	20.2 ± 13.3	0.49
Physical activity (minutes/week)	402.9 ± 128.3	356.7 ± 218.1	0.63
Healthy Eating Index (HEI)	43.5 ± 10.6	51.9 ± 17.8	0.28

Data are represented as mean ± SD.

**Table 4:** Effects of the five-week soy and lupin intervention on biochemical, anthropometric, and metabolic parameters of adolescents with overweight and obesity.

	Soy			Lupin		
	Baseline	5 Weeks		Baseline	5 Weeks	
	Mean ± SD	Mean ± SD	*p*	Mean ± SD	Mean ± SD	*p*
**Biochemical**						
Glucose (mg/dL)	**99.5 ± 6.55**	93.1 ± 6.54 **	**0.01**	97.9 ± 9.54	92.3 ± 5.44	0.08
Insulin (μU/mL)	14.8 ± 19.78	8.78 ± 4.44	0.20	11.5 ± 10.51	9.0 ± 5.44	0.16
C-peptide (pg/mL)	**1137.2 ± 636.8**	933.3 ± 398.03 *	**0.03**	**1018.1 ± 505.31**	855.4 ± 311.57 *	**0.05**
Glucagon (pg/mL)	3746.8 ± 583.68	3599.8 ± 567.39	0.16	**3682.0 ± 398.6**	3331.4 ± 300.99 ***	**0.001**
Triglycerides (mg/dL)	**90.86 ± 43.94**	75.29 ± 36.96 *	**0.02**	98.8 ± 89.98	98.5 ± 109.22	0.98
Total Cholesterol (mg/dL)	**157.3 ± 45.33**	141.0 ± 33.05 *	**0.03**	145.7 ± 24.49	147.2 ± 23.81	0.44
c-HDL (mg/dL)	37.5 ± 7.35	36.93 ± 6.81	0.61	33.9 ± 7.99	35.7 ± 12.15	0.35
c-LDL (mg/dL)	111.6 ± 45.38	102.9 ± 30.26	0.20	**103.7 ± 27.11**	**112.3 ± 28.02**	**0.002**
Urea (mg/dL)	**46.9 ± 8.29**	37.6 ± 5.39 **	**0.01**	**41.6 ± 9.93**	30.9 ± 6.44 ***	**0.001**
Creatinine (mg/dL)	0.87 ± 0.09	0.82 ± 0.25	0.44	**0.92 ± 0.14**	0.75 ± 0.24 *	**0.003**
AST (U/L)	36.3 ± 9.28	34.3 ± 7.01	0.34	30.3 ± 7.51	30.4 ± 6.24	0.95
ALT (U/L)	26.8 ± 13.44	29.3 ± 14.11	0.28	**25.8 ± 7.84**	30.4 ± 8.84 ***	**0.001**
**Metabolic**						
Leptin (pg/mL)	4664.9 ± 3491.6	4165.5 ± 2619.1	0.30	3563.8 ± 2589.1	2844.0 ± 1535.9	0.11
Adiponectin (pg/mL)	65,159.2 ± 3122	64,155.8 ± 3541.2	0.14	64,988.7 ± 1797.5	65,277.5 ± 2108.1	0.71
HOMA2-IR	**0.88 ± 0.50**	0.74 ± 0.28 *	**0.02**	**0.85 ± 0.37**	0.69 ± 0.23 **	**0.01**
HOMA2-%B	69.22 ± 25.0	71.0 ± 18.4	0.70	72.5 ± 35.8	69.24 ± 14.7	0.77
HOMA2-%S	**140.7 ± 59.2**	153.7 ± 54.4 **	**0.005**	**141.9 ± 62.1**	161.0 ± 51.9 *	**0.05**
**Anthropometric**						
Weight (kg)	**73.4 ± 11.98**	74.49 ± 11.89 *	**0.02**	71.7 ± 8.80	71.9 ± 9.66	0.70
Height (m)	**1.63 ± 0.06**	1.64 ± 0.07 ***	**0.001**	**1.63 ± 0.07**	1.64 ± 0.07 **	**0.003**
BMI for age Z-score	2.2 ± 0.5	2.3 ± 0.5	0.11	2.2 ± 0.4	2.1 ± 0.5	0.23
Height for age Z-score	0.16 ± 0.88	0.06 ± 0.83	0.24	0.003 ± 0.9	0.11 ± 0.92	0.22
Body fat percentage	26.0 ± 5.59	27.5 ± 9.21	0.26	24.4 ± 5.37	24.7 ± 4.67	0.63
Waist circumference	88.6 ± 10.39	88.5 ± 8.86	0.85	85.2 ± 5.91	85.41 ± 6.11	0.63

Results are presented as mean ± SD. Comparison between five weeks after soy and lupin intervention versus baseline was performed by Student’s *t*-test. * *p* ≤ 0.05, ** *p* ≤ 0.01, *** *p* ≤ 0.001 are indicated in bold letters. SB, *n* = 15; LB *n* = 15.

## Data Availability

The original contributions presented in this study are included in the article/Supplementary Materials. Further inquiries can be directed to the corresponding author.

## Supplementary Materials

The following supporting information can be downloaded at: https://www.mdpi.com/article/10.3390/nu18132049/s1, Table S1: Baseline distribution of participants according to weight status (overweight and obesity) in the soy-beverage (SB) and lupin-beverage (LB) groups Table S2: Comparison of clinical parameters between LB and SB groups at the basal state; Table S3: Comparison of changes after intervention in biochemical, anthropometric, and metabolic parameters.
