# Supplementary material for: Functional Soy and Lupin Protein-Based Beverages Modulate Gut Microbiome and Attenuate Metabolic Dysregulation in Adolescent Boys with Overweight and Obesity
Source: Nutrients. 2026 Jun 23;18(13):2049. doi: 10.3390/nu18132049 (PMC13363434; doi:10.3390/nu18132049)
Supplement: Supplementary file 1 [file nutrients-18-02049-s001.zip › nutrients-4324547-supplementary.pdf]

Table S1. Baseline distribution of participants according to weight status (overweight and obesity) in the soy-beverage (SB) and lupin-beverage (LB) groups.

Weight Status	SB (*n*=15)	LB (*n*=15)	*p* value
Overweight	8	7	
Obesity	7	8	>0.9999

Values represent the *n* for each category. Differences in the distribution of overweight and obesity between the SB and LB groups were assessed using Fisher's exact test. No significant differences were observed between groups (p > 0.9999).

Table S2. Comparison of clinical parameters between LB and SB groups at the basal state.

	SB	LB	*p*
**Parameters**			
Glucose (mg/dl)	99.5 ± 6.55	97.9 ± 9.54	0.55
Insulin (mU/ml)	14.8 ± 19.78	11.5 ± 10.51	0.61
C-peptide (pg/ml)	1137.2 ± 636.8	1018.1 ± 505.31	0.82
Glucagon (pg/ml)	3746.8 ± 583.68	3682.0 ± 398.6	0.80
Tryglycerides (mg/dl)	90.86 ± 43.94	98.8 ± 89.98	0.81
Total cholesterol (mg/dl)	157.3 ± 45.33	145.7 ± 24.49	0.32
c-HDL (mg/dl)	37.5 ± 7.35	33.9 ± 7.99	0.12
c-LDL (mg/dl)	111.6 ± 45.38	103.7 ± 27.11	0.54
Urea (mg/dl)	46.9 ± 8.29	41.6 ± 9.93	0.10
Creatinine (mg/dl)	0.87 ± 0.09	0.92 ± 0.14	0.36
AST (IU/L)	36.3 ± 9.28	30.3 ± 7.51	0.11
ALT (IU/L)	26.8 ± 13.44	25.8 ± 7.84	0.92
HOMA-IR	0.85 ± 0.37	0.88 ± 0.50	0.57
Leptin (pg/ml)	4664.9 ± 3491.6	3563.8 ± 2589.1	0.33
Adiponectin (pg/ml)	65159.2 ± 3122	64988.7 ± 1797.5	0.77
PAI-1 (pg/ml)	22513 ± 5291	22151 ± 5557	0.86
Resistin (pg/ml)	4822 ± 2907	6345 ± 3739	0.23
Visfatin (pg/ml)	5322 ± 683.5	5337 ± 640.3	0.95
Ghrelin (pg/ml)	**151.8 ± 28.52**	**206.2 ± 91.39**	**0.04**
GIP (pg/ml)	**58.06 ± 12.32**	**95.56 ± 41.48**	**0.003**
GLP-1 (pg/ml)	103.9 ± 22.77	110.7 ± 24.04	0.43
Weight (Kg)	73.4 ± 11.98	71.7 ± 8.80	0.55
Height (m)	1.63 ± 0.06	1.63 ± 0.07	0.62
BMI for age Z-score	2.2 ± 0.5	2.2 ± 0.4	0.85
Height for age Z-score	0.16 ± 0.88	0.003 ± 0.9	0.40
Waist circumference	88.6 ± 10.39	85.2 ± 5.91	0.36
Body fat percentage	26.0 ± 5.59	24.4 ± 5.37	0.63

Results are presented as mean ± SD. Comparison between soy and lupin at baseline was performed by Student's *t*-test. * $p \leq 0.05$, ** $p \leq 0.01$ are indicated in bold letters. SB, $n = 15$; LB $n = 15$.

Table S3. Comparison of changes after intervention in biochemical, anthropometric, and metabolic parameters.

	(post - pre) SB	(post - pre) LB	*p*
**Parameters**			
Glucose (mg/dl)	-6.4 ± 7.99	-5.6 ± 11.12	0.83
Insulin (mU/ml)	-6.02 ± 16.51	-2.5 ± 6.21	0.51
C-peptide (pg/ml)	-203.9 ± 316.02	-162.6 ± 287.31	0.72
Glucagon (pg/ml)	-146.95 ± 372.62	-350.63 ± 235.22	0.10
Tryglycerides (mg/dl)	-15.57 ± 22.39	-0.3 ± 32.94	0.16
Total cholesterol (mg/dl)	**-16.3 ± 25.39**	**1.5 ± 7.07**	**0.02**
c-HDL (mg/dl)	-0.57 ± 4.09	1.9 ± 7.10	0.26
c-LDL (mg/dl)	**-8.7 ± 24.06**	**8.6 ± 8.05**	**0.02**
Urea (mg/dl)	-9.3 ± 10.83	-10.7 ± 9.51	0.72
Creatinine (mg/dl)	-0.05 ± 0.23	-0.17 ± 0.18	0.14
AST (IU/L)	-2 ± 7.62	0.1 ± 7.71	0.47
ALT (IU/L)	2.5 ± 8.23	4.6 ± 3.74	0.39
HOMA2-IR	-0.14 ± 0.28	-0.16 ± 0.25	0.92
Leptin (pg/ml)	-499.38 ± 1733.21	-719.8 ± 1432.0	0.72
Adiponectin (pg/ml)	-1003.4 ± 2314.7	288.8 ± 2779.3	0.19
PAI-1 (pg/ml)	-4025.2 ± 4464.2	-3301.2 ± 2699.5	0.64
Resistin (pg/ml)	-748.69 ± 1682.6	-1988.33 ± 3248.8	0.21
Visfatin (pg/ml)	-698.6 ± 940.1	-495.2 ± 651.9	0.50
Ghrelin (pg/ml)	**2.24 ± 17.67**	**-35.88 ± 47.03**	**0.009**
GIP (pg/ml)	**-1.42 ± 19.48**	**-21.43 ± 28.10**	**0.04**
GLP-1 (pg/ml)	-3.01 ± 15.53	-3.38 ± 15.55	0.95
Weight (Kg)	1.11 ± 1.07	0.22 ± 2.19	0.18
Height (m)	0.01 ± 0.005	-0.01 ± 0.008	0.69
BMI for age Z-score	0.06 ± 0.13	-0.07 ± 0.22	0.06
Height for age Z-score	0.11 ± 0.33	-0.10 ± 0.33	0.09
Waist circumference	-0.14 ± 2.59	0.21 ± 1.65	0.67
Body fat percentage	1.5 ± 4.77	0.3 ± 1.94	0.35

Results are presented as mean ± SD. The intervention effect was calculated as the change from baseline (Δ= post-intervention – pre-intervention). Comparisons of Δ values between the soy (SB) and lupin (LB) groups were performed using Student's t-test. Values shown in bold indicate statistically significant differences between groups, * $p \leq 0.05$, ** $p \leq 0.01$. SB, $n = 15$; LB $n = 15$.
